# Supplementary material for: Comparison of methods for rhythm analysis of complex animals’ acoustic signals
Source: PLoS Comput Biol. 2020 Apr 8;16(4):e1007755. doi: 10.1371/journal.pcbi.1007755 (PMC7141653; doi:10.1371/journal.pcbi.1007755)
Supplement: S1 Table — (DOCX) [file pcbi.1007755.s001.docx]

**S1 Table**

**Comparison of methods for rhythm analysis of complex animal vocalizations**

Lara S. Burchardt*, Mirjam Knörnschild

^*^ Corresponding author: l.s.burchardt@gmx.de

**Validation normalized Goodness-of-Fit value, Fourier analysis**

To validate the use of the normalized goodness-of-fit value (nGOF) in Fourier analysis, we correlate it with the sample length, to show that the nGOF shows the smallest correlation coefficient as compared to the goodness-of-fit value that was not normalized (GOF) or the amplitude in the frequency spectrum. These correlations only show when including all analysed data, ranging from very short to very long sequences. To ensure comparability between exactly these very different sequences, it is important to use the value least correlated to the sample length.

Furthermore, we validated the nGOF by comparing and correlating it to the already published goodness-of-fit value for the Generate-and-Test approach, i.e. the frequency-normalized root-mean-square-deviation (FRMSD). The two values strongly correlate, which ensures us it is appropriate to use it as a goodness-of-fit value. All correlation coefficients were calculated in R (version 3.5.3) with the function ‘cor’, which by default is calculating a Pearson correlation. We show the results in the table.

**S1 Table 1: Correlation of goodness-of-fit and related values**

|  | nGOF | GOF | P | sample length | FRMSD | # elements |
| --- | --- | --- | --- | --- | --- | --- |
| nGOF |  |  |  |  |  |  |
| GOF | 0.73 |  |  |  |  |  |
| P | 0.94 | 0.83 |  |  |  |  |
| sample length | -0.48 | -0.78 | -0.66 |  |  |  |
| FRMSD | -0.77 | -0.84 | -0.86 | 0.71 |  |  |
| # elements | -0.51 | -0.79 | -0.69 | 0.99 | 0.73 |  |
