# Supplementary material for: Comparison of methods for rhythm analysis of complex animals’ acoustic signals
Source: PLoS Comput Biol. 2020 Apr 8;16(4):e1007755. doi: 10.1371/journal.pcbi.1007755 (PMC7141653; doi:10.1371/journal.pcbi.1007755)
Supplement: S2 Table — (DOCX) [file pcbi.1007755.s002.docx]

**S2 Table**

**Comparison of methods for rhythm analysis of complex animal vocalizations**

Lara S. Burchardt*, Mirjam Knörnschild

^*^ Corresponding author: l.s.burchardt@gmx.de

**Artificial data**

To make it easier to set results into perspective, we analysed three artificial data sets, using the same workflow as for the original biological data.

Results were calculated for perfect isochronous sequences with Inter-Onset-Intervals (IOIs) of 0.1, 0.3 and 0.5 seconds. In a second dataset numbers were drawn randomly from a uniform distribution ranging from 0 to 1. A third dataset consisted of numbers randomly drawn from a Gaussian distribution with three different parameter combinations. 100 elements per sequence, 10 sequences each were drawn for the following parameter combinations: 1) mean: 0.1 seconds, standard deviation: 0.05 seconds; 2) mean: 0.2 seconds, standard deviation: 0.1 seconds and 3) mean: 1 second, standard deviation: 0.5 seconds. That way, we expected results to be around 10 Hz, 5 Hz and 1 Hz respectively for Inter-Onset-Interval analysis, since this simply depends on the mean. The coefficient of variation (C_v_) should be very similar between the three groups, since the relation between mean and standard deviation was chosen to be the same in all three cases. That way, the difference between the mean coefficient of variation for all sequences and the coefficient of variation overall over different subsets of the data illustrates the relation between the difference between the two and the possibility of different underlying beats very nicely. Different beats, analysed separately, can have the same C_v_ mean for all sequences, since the variation is similar in all the sequences (as the modelled relation between mean and standard deviation is similar). As we calculate the results over the different subsets combined, the variation increases, because Inter-Onset-Intervals from the different modelled distributions form the basis for the calculation and therefore the variation increases. If we transfer that to our original data sets this means that the higher the difference between the two values, the higher the probability that different sub distributions underlie the different sequences. That indicates differences between sequences and/or individuals. All results are shown in the table, where the first given value represents the expected value, whereas the second value is the actually calculated value. They fit very nicely in all cases.

**S2 Table : IOI analysis of artificial data, expected values and calculated results**

| Dataset | Mean[sec] | Std [sec] | C_v_ mean of sequences | C_v_ overall | Beat [Hz] |
| --- | --- | --- | --- | --- | --- |
| 1 Hz | 1/1.05 | 0.5/0.48 | 0.45/0.45 | 0.45/0.46 | 1 / 0.96 |
|  |  |  |  |  |  |
| 5 Hz | 0.2/0.21 | 0.1/0.1 | 0.45/0.46 | 0.45/0.46 | 5 / 4.78 |
| 10 Hz | 0.1/0.11 | 0.05/0.05 | 0.45/0.46 | 0.45/0.46 | 10 / 9.57 |
| 1 Hz + 5 Hz | 0.6 /0.63 | 0.5 / 0.54 | 0.45/0.46 | -- / 0.86 | 1.7 / 1.6 |
| 1 Hz + 10 Hz | 0.55/0.57 | 0.5 / 0.58 | 0.45/46 | -- / 1.01 | 1.8 / 1.74 |
| 5 Hz + 10 Hz | 0.15/0.16 | 0.1 / 0.1 | 0.45/46 | -- / 0.58 | 6.7 / 6.4 |
| 1 Hz + 5 Hz + 10 Hz | 0.44 /0.45 | 0.5 / 0.5 | 0.45/46 | -- / 1.18 | 2.3/ 2.21 |
