# Supplementary material for: Comparison of methods for rhythm analysis of complex animals’ acoustic signals
Source: PLoS Comput Biol. 2020 Apr 8;16(4):e1007755. doi: 10.1371/journal.pcbi.1007755 (PMC7141653; doi:10.1371/journal.pcbi.1007755)
Supplement: S3 Table — (DOCX) [file pcbi.1007755.s003.docx]

**S3 Table**

**Comparison of methods for rhythm analysis of complex animal vocalizations**

Lara S. Burchardt*, Mirjam Knörnschild

^*^ Corresponding author: l.s.burchardt@gmx.de

**Rhythm analysis results of artificial data**

Here we show the resulting exact beats for the three different methods Inter-Onset-Interval analysis, Fourier analysis and the Generate-and-test approach. We show the detailed numbers for the Fourier analysis, to give insight into what the newly established goodness-of-fit values looks like for different datasets (S1 Table 3-5). Furthermore, we show results for all methods in a histogram depiction for the datasets drawn from normally distributed numbers (S1 Figure 1). Furthermore we show the results of the cluster analysis on dataset 3, drawn from three Gaussian distributions (S1 Figure 2). We can see very clear clusters for the IOI analysis, as these are based on the modelled means, but for the other two methods we do not see clear clusters at all. This is especially interesting as in our original datasets we see in parts similar coefficients of variation, the resulting clusters are still much stronger (20-30% in one cluster in artificial data versus 33-82% in biological data for the GAT approach for example). Also the expected beat frequencies, based on the modeled distributions would be 1 Hz for sub-dataset 1, 5 Hz for sub-dataset 2 and 10 Hz for sub-dataset 3. This is not at all supported by the results, which again shows that Inter-Onset-Interval analysis oversimplifies results considerably.

**S3 Table: Results of rhythm analysis for artificial data**

| **Results of Fourier analysis for perfectly isochronous sequences of different Inter-Onset-Intervals** | | | | | |
| --- | --- | --- | --- | --- | --- |
| Modelled IOI | Amplitude P | Resulting beat [Hz] | Sample length | GOF | nGOF |
| 0.1 sec | 0.0503 | 9.995 | 1981 | 0.9958 | 5.0268e-04 |
| 0.3 sec | 0.0168 | 3.333 | 5941 | 0.9995 | 1.6824e-04 |
| 0.5 sec | 0.0101 | 1.9998 | 9901 | 0.9998 | 1.0098e-04 |
|  | | | | | |
| **Results of Fourier analysis for sequences drawn from a uniform distribution between 0 and 1** | | | | | |
| Sequence # | Amplitude P | Resulting beat [Hz] | Sample length | GOF | nGOF |
| 1 | 0.0031 | 54.49 | 10398 | 0.3180 | 3.0578e-05 |
| 2 | 0.0031 | 91.67 | 10045 | 0.3105 | 3.0912e-05 |
| 3 | 0.0032 | 82.06 | 9318 | 0.2937 | 3.1516e-05 |
| 4 | 0.0032 | 24.62 | 9961 | 0.3200 | 3.2129e-05 |
| 5 | 0.0029 | 46.45 | 9555 | 0.2753 | 3.8814e-05 |
| 6 | 0.0029 | 88.89 | 9774 | 0.2856 | 3.9225e-05 |
| 7 | 0.0031 | 32.91 | 9906 | 0.3119 | 3.1488e-05 |
| 8 | 0.0030 | 47.43 | 10196 | 0.3098 | 3.0384e-05 |
| 9 | 0.0031 | 81.36 | 8987 | 0.2793 | 3.1074e-05 |
| 10 | 0.0038 | 15.96 | 8483 | 0.3244 | 3.8238e-05 |
|  |  |  |  |  |  |
| **Results of Fourier analysis for sequences drawn from three different Gaussian distributions** | | | | | |

| Sequence # | Amplitude P | Resulting beat [Hz] | Sample length | GOF | nGOF |
| --- | --- | --- | --- | --- | --- |
| mean_1_seq01 | 0.0014718 | 3.40623546 | 21490 | 0.31948573 | 1.4867E-05 |
| mean_1_seq02 | 0.00136936 | 26.9280919 | 20707 | 0.28355351 | 1.3694E-05 |
| mean_1_seq03 | 0.00129352 | 37.3855158 | 22383 | 0.28952889 | 1.2935E-05 |
| mean_1_seq04 | 0.00144668 | 60.5123294 | 20885 | 0.30213879 | 1.4467E-05 |
| mean_1_seq05 | 0.00158602 | 20.1604867 | 19067 | 0.30240621 | 1.586E-05 |
| mean_1_seq06 | 0.00150281 | 19.8842456 | 20388 | 0.30639232 | 1.5028E-05 |
| mean_1_seq07 | 0.00147356 | 4.13854704 | 20152 | 0.29695108 | 1.4736E-05 |
| mean_1_seq08 | 0.00145706 | 21.9145803 | 20370 | 0.29680217 | 1.4571E-05 |
| mean_1_seq09 | 0.00153698 | 74.6243407 | 20098 | 0.30890217 | 1.537E-05 |
| Mean_1_seq10 | 0.00142628 | 71.777362 | 21380 | 0.3049378 | 1.4263E-05 |
| mean_0.2_seq01 | 0.00649635 | 17.0134073 | 4326 | 0.28103226 | 6.4964E-05 |
| mean_0.2_seq02 | 0.00602181 | 97.4408498 | 4142 | 0.24942354 | 6.0218E-05 |
| mean_0.2_seq03 | 0.00656363 | 34.7476552 | 4478 | 0.29391954 | 6.5636E-05 |
| mean_0.2_seq04 | 0.00678646 | 7.03685974 | 4178 | 0.28353816 | 6.7865E-05 |
| mean_0.2_seq05 | 0.00783899 | 47.5616151 | 3814 | 0.29897908 | 7.839E-05 |
| mean_0.2_seq06 | 0.00711769 | 14.9092692 | 4078 | 0.29025924 | 7.1177E-05 |
| mean_0.2_seq07 | 0.00716861 | 42.2227735 | 4031 | 0.28896665 | 7.1686E-05 |
| mean_0.2_seq08 | 0.00641353 | 74.3067485 | 4075 | 0.26135143 | 6.4135E-05 |
| mean_0.2_seq09 | 0.00704148 | 6.41631435 | 4021 | 0.28313806 | 7.0415E-05 |
| mean_0.2_seq10 | 0.00668986 | 35.9214219 | 4276 | 0.28605838 | 6.6899E-05 |
| mean_0.1_seq01 | 0.01192391 | 19.4085028 | 2164 | 0.25803339 | 0.00011924 |
| mean_0.1_seq02 | 0.01399666 | 72.4903475 | 2072 | 0.29001089 | 0.00013997 |
| mean_0.1_seq03 | 0.01315409 | 69.4953104 | 2239 | 0.29452001 | 0.00013154 |
| mean_0.1_seq04 | 0.01288567 | 14.0737195 | 2089 | 0.26918168 | 0.00012886 |
| mean_0.1_seq05 | 0.01582618 | 35.8678553 | 1907 | 0.30485385 | 0.00015986 |
| mean_0.1_seq06 | 0.01279744 | 29.8039216 | 2040 | 0.26106775 | 0.00012797 |
| mean_0.1_seq07 | 0.01363039 | 41.3690476 | 2016 | 0.27478874 | 0.0001363 |
| mean_0.1_seq08 | 0.01276541 | 21.5897939 | 2038 | 0.260159 | 0.00012765 |
| mean_0.1_seq09 | 0.01502785 | 55.2238806 | 2010 | 0.30205979 | 0.00015028 |
| mean_0.1_seq10 | 0.01458886 | 91.8186068 | 2139 | 0.31205579 | 0.00014589 |
