# Supplementary material for: Comparison of methods for rhythm analysis of complex animals’ acoustic signals
Source: PLoS Comput Biol. 2020 Apr 8;16(4):e1007755. doi: 10.1371/journal.pcbi.1007755 (PMC7141653; doi:10.1371/journal.pcbi.1007755)
Supplement: S1 Fig — (DOCX) [file pcbi.1007755.s004.docx]

**S1 Figure**

**Comparison of methods for rhythm analysis of complex animal vocalizations**

Lara S. Burchardt*, Mirjam Knörnschild

**
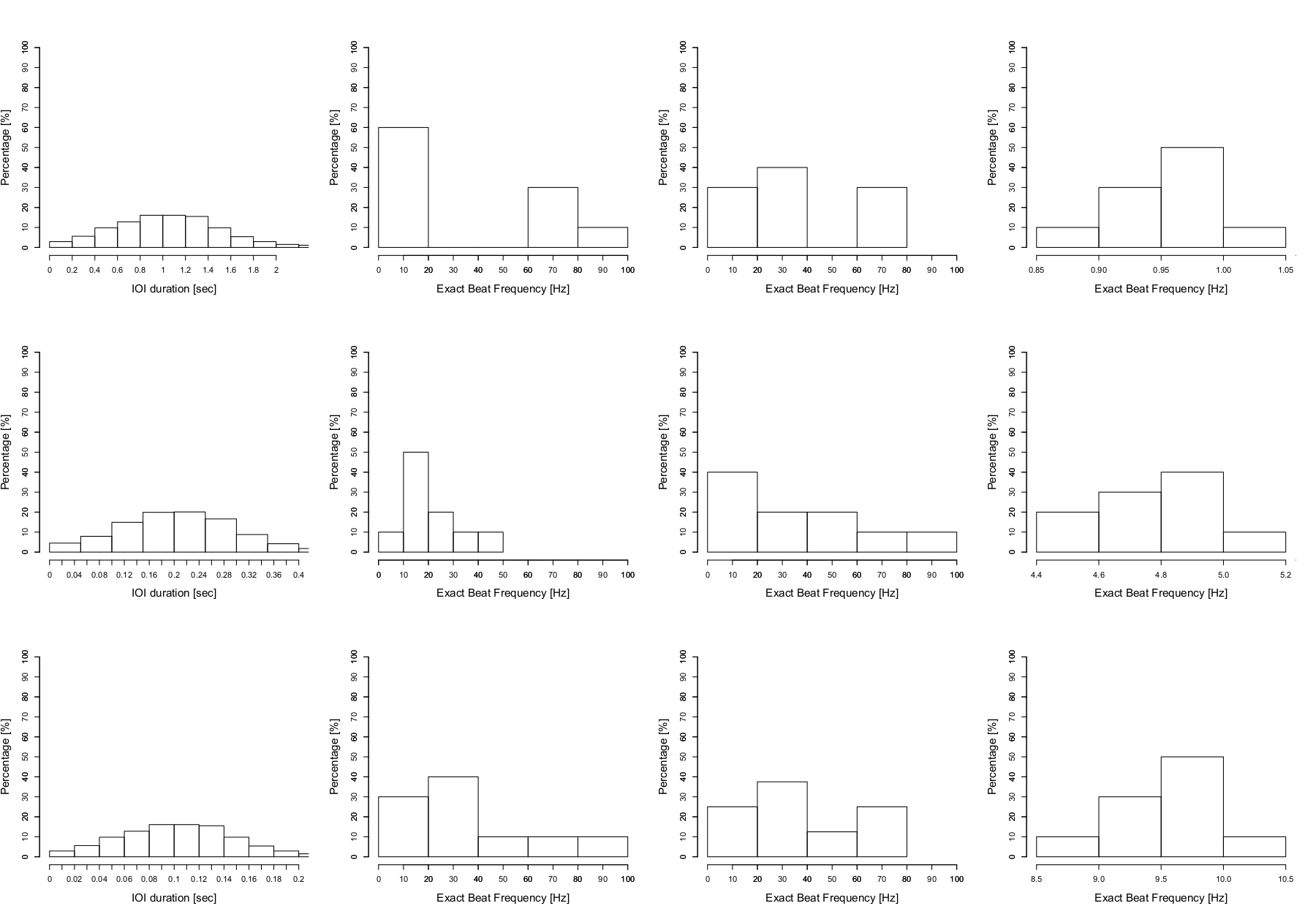
**^*^ Corresponding author: [l.s.burchardt@gmx.de](mailto:l.s.burchardt@gmx.de)

**S1 Figure: Results of Rhythm analysis: drawn from Gaussian distributions with differing means were three datasets (means of datasets from row 1 to 3: 1 sec, 0.2 sec, 0.1 sec respectively). The first column depicts the distribution of Inter-Onset-Intervals in the 10 sequences (1000 elements) the second to fourth column depict the analyzed exact beat frequencies found with: a Generate-end-test approach, Fourier Analysis and Inter-Onset-Interval analysis.**
