# Supplementary material for: Comparison of methods for rhythm analysis of complex animals’ acoustic signals
Source: PLoS Comput Biol. 2020 Apr 8;16(4):e1007755. doi: 10.1371/journal.pcbi.1007755 (PMC7141653; doi:10.1371/journal.pcbi.1007755)
Supplement: S2 Fig — (DOCX) [file pcbi.1007755.s005.docx]

**S2 Figure**

**Comparison of methods for rhythm analysis of complex animal vocalizations**

Lara S. Burchardt*, Mirjam Knörnschild

**
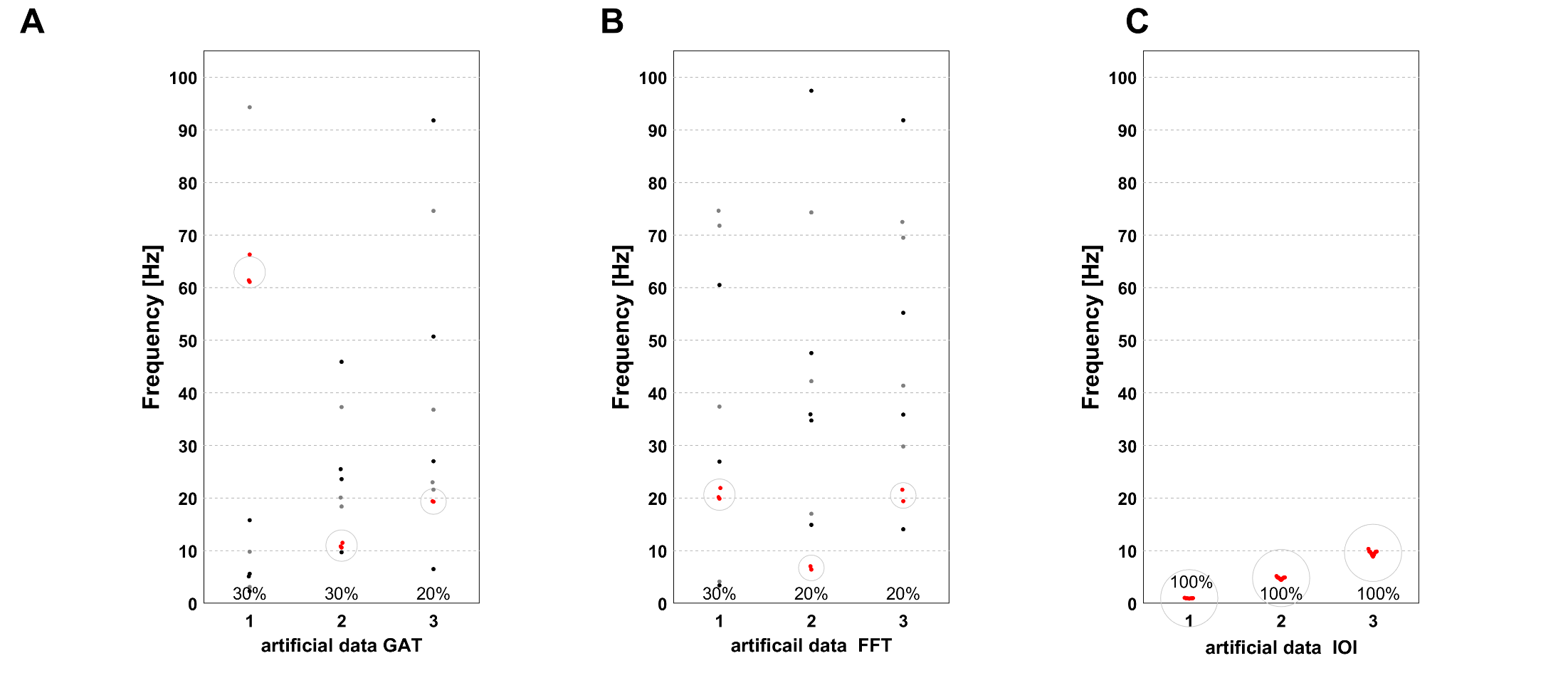
**^*^ Corresponding author: [l.s.burchardt@gmx.de](mailto:l.s.burchardt@gmx.de)

**S2 Figure: Cluster analysis of artificial data (A) results for the GAT approach, (B) results for the Fourier analysis, (C) results for the Inter-Onset-Interval analysis. The numbers on the bottom indicate the sub dataset. ‘1’ stands for the dataset drawn from a distribution with a mean of 1 with a SD of 0.5, ‘2’ is the dataset with a mean of 0.2 seconds and SD of 0.1 and ‘3’ is the dataset with a mean of 0.1 seconds and SD of 0.05.**
