# Supplementary material for: Comparison of methods for rhythm analysis of complex animals’ acoustic signals
Source: PLoS Comput Biol. 2020 Apr 8;16(4):e1007755. doi: 10.1371/journal.pcbi.1007755 (PMC7141653; doi:10.1371/journal.pcbi.1007755)
Supplement: Attachment — Submitted filename: Response to Reviewer_Burchardt_PCOMPBIOL-D-19-01995.docx [file pcbi.1007755.s007.docx]

Response to Reviewer Comments:

**We thank two anonymous reviewers for their time and helpful suggestions. The reviewers’ comments were greatly appreciated and we have followed their suggestions whenever possible. When we have not followed a suggestion, we have provided a justification for doing so. Below, we describe in detail how we have revised the manuscript by addressing each suggestion/comment separately. Line numbers refer to the revised manuscript. Changes in the revised manuscript are highlighted.**

Reviewer #1: In this manuscript, the authors showcase both old and new methods to test for the presence of temporal regularities in animal sounds. The ms is timely, well-written and sound, and it will be very helpful to a number of scientists. I was very happy to read this important piece of work.

I have a couple of major comments and several minor ones (see below).
1) Focus: The title, abstract and story of the ms are heavily centered on rhythm. This is great but the analysis methods and pipeline seem to me more about isochrony and periodicities rather than rhythm per se. A slight refocus (still keeping the rhythm, big picture in mind) may be good.

**Thank you very much for this observation. We refocused according to the reviewer’s suggestions. We explain now what we mean when using the term ‘rhythm’ using it as a concept everyone understands rather than a technical term (lines 56-61). We now also explain key terms with regards to rhythm such as periodicity in a newly created glossary (line 105). We agree that the study rather investigates periodicity and isochrony, which we now state more clearly at different places (e.g. lines 85 – 94, lines 139-140). We hope these changes make it more clear what we are actually describing.**

2) Baseline and validation: Some of the (mathematical) claims do not appear fully supported by the available text. I am sure the authors thought about this topic thoroughly, but if this is to become ‘the’ method for spotting/measuring temporal regularities in animal sounds, the maths/stats behind it need to be solid. Therefore, and practically, I’d encourage the authors to 1) make sure all the important claims and choices they make are fully supported by the maths and previous literature (see ‘detailed issues’ below for some specific points); 2) run the analyses also with some ‘synthetic’ temporal sequences, e.g. a perfectly isochronous one, a random sequence sampled from a uniform distribution, a random sequence sampled from a normal distribution or pink noise, etc.

**This is a very important point, and we are happy to provide more information than before.** **It appears one of the key concerns here is for example the missing validation of the goodness-of-fit values for the Fourier analysis. We are happy to provide these in the S1 Supporting Information in detail. To justify the use of the nGOF over other parameters, we show the correlation coefficients with values such as sample length or number of elements. These shouldn’t be correlated with the nGOF to make the parameter worth using in comparative approaches. As you will find, the correlation between nGOF and the parameter of interest is the lowest compared to GOF or the simple Fourier signal amplitude. Furthermore we validated it by correlating it with the goodness-of-fit value of the GAT approach that was already established (Norton & Scharff, 2016; Burchardt et al, 2019). They show a high correlation (-0.77). All these values are now shown in the S1 Supporting Information. Also we included artificial data into the manuscript. Now we can show some theoretical examples, as well as explain through example how C_V_ (overall) and C_V_ (mean of sequences) are related. Since a paper showing the results for theoretical values already exists (Ravignani & Norton, 2017), we ran the analysis only for a subset of the suggested examples, ones we thought would illustrate the innovative points best. To be able to illustrate the results and their meaning best, we chose to model three datasets drawn from Gaussian distributions with different means (i.e. resulting in different beat frequencies) but similar variation (see line 267-277 and S1). We also show some of the parameters for a prefect isochronous pattern and a dataset drawn at random from a uniform distribution ranging from 0 to 1.**

Minor/detailed issues:
Title: Is ‘Method comparison’ correct in English?

**The title was changed to ‘Comparison of methods’, thank you.**
line 14: an ‘and’ may be needed before ‘ecologists’ **Done.**27: This sentence is a bit awkward. **Thank you, we rephrased the sentence. (lines 27-28)**
37: What is the meaning of ’highly computational’? and can a FFT be considered such thing?

**We rephrased that to “higher mathematics”**, **since “highly computational is indeed misleading and the FFTs used might not be considered “highly computational”. (line 38)**

68-69 is this definition of animal sound broadly shared?

**This is not supposed to be a general definition, but supposed to correctly name whale sounds, since these are no vocalizations, as they are not produced by vocal cords. We were made aware of that by colleagues doing research in whale bioacoustics and therefore added this**. **As mentioned before, we added a glossary which also includes this explanation (line 105).**

70: did the authors mean ‘connotations’? **Yes, thank you. Changed.**
120-121: Do the authors agree that rhythmic =/= periodic? In any case, how do they define periodic?

**We do agree on that. We define that a sequence is periodic, when it has an underlying isochronous structure, where not all “notes” or “beats” necessarily need to be present. We now state that in the manuscript and in the glossary, thank you for pointing that out.** **(line 82-94, line 105)**
127-128: How do the authors define ‘beat’ here? **We define ‘beat’ in the glossary now as the unit to describe an isochronous pattern (lines 90-91, line 105)**
137: Why ‘IOI-‘ and not ‘IOI’? **Typo, thank you, changed.**
174: Is ‘please’ necessary? **Probably not, deleted.**
179-onwards: The ‘rhythm analyses’ section is a bit messy, mixing here and there definitions with explanations of concepts and actual methods/analyses/choices made in this paper.

**Since the methods are the focus of this paper, it felt right to justify some choices directly, but we agree that it is messy and not best practice. Therefore, we added ‘discussion of methods’ to the results section and transferred explanations to the introduction (i.e. lines 133-134) and choices and justification into the new discussion section (e.g. lines 295-300, 339-353, 383-384, 396-403, 473-476). We believe that this resulted in a more coherent form of the manuscript.**
187-188: Please check verbal tense agreement, here and elsewhere. **We re-read the manuscript and adjusted verbal tenses to the best of our knowledge.** **(e.g. lines 217, 226, 239, 242)**
190: What is a ‘rhythm frequency’?

**Terms got mixed up here. We now consistently use the term “exact beat frequency”, which is explained in the glossary as ‘the beat frequency we calculated to describe a specific sequence best (i.e. 5 Hz as in 5 beats per second)’.**
191: What is a ‘consistent rhythm’? **It was meant to refer to the variability of a particular sequence, the term ‘consistent rhythm’ is unclear and misleading and was therefore deleted and the more clear wording used. (line 219)**
195-196: Is there an approximate/suggested cutoff value on n for using eq. (1) vs. (2)?

**To the best of our knowledge we cannot provide a clear cutoff, but looking at our data, biased and unbiased estimator are getting closer to being similar from an n of ~20. Since the unbiased estimator is more conservative, we chose to use this even for very short sequences.**

198: sizE **Thank you, added.**
200: nPVI: Is this really the case? Why? **It is easiest to understand it as such, but we changed the formulation to the more precise version, that it is a measure of variability (line 134).**
208: interval (only one ’l’) **Changed, thank you.**
209: A plot…is a possibility’? **This was a wrong formulation, thank you for pointing that out. The sentence was restructured. (lines 235-236)**
234: add, e.g. ‘in the past’? **We added ‘so far’ (line 399), thank you.**
240: consider replacing with ’but is, instead, the mean’. **Yes, this makes it more clear. Thank you (line 247).**
249: Euler with capital ‘E’? **Yes, thank you. Changed. (line 256)**
252-253: This statement should be proven mathematically. **The correlations are now shown in the Supporting Information. We are sorry to have not provided that information before.**
260: what is the ‘respected…window’?

**Sorry, we did not correctly adjust that after changes in the manuscript. Thank you for pointing that out. The frequency window analysed is 2 – 100 Hz, which we now also state in the text. (line 264)**
288-289: Is the Cv normally distributed? If not, how is it distributed across bouts?

**The Cv is not normally distributed (Shapiro Wilk, W = 0.88211, p-value = 7.059e-10). The distribution differs between the species, the Cv values in the datasets can best be described as Poisson like.**

Table 1: What is the meaning of the ‘crossed 0’ symbol? Isn’t it often used to denote the empty set?

**The crossed zero is the sign for “average” or “mean”, since this seems to be not distinct, we changed that to “mean”. Thank you for pointing out this misleading use of symbols.**
325-327: This sentence is unclear. Also, and I am sorry if I am missing something, but I don’t see how the next sentence is linked and/or opposed to this sentence. **We rephrased that sentence, hopefully reducing ambiguity and added a transition to the next sentence, to clear things up.** (line 334-336)
456: ‘to analyze’-> ‘analyzed’ **Changed.**
460 and elsewhere, including the title: ‘periodicity and rhythmicality’ I believe this paper is rather about ‘periodicity and isochrony’, and rhythm plays a more indirect role

**As we answered to your major concerns, we tried rephrasing and clearing this up in the manuscript as much as possible, but keeping the phrase “rhythmicity” as a concept everyone understand. We hope we demonstrated adequately that we are aware of the phrasing issue. (i.e. lines: 51-52,56-61, 85-94, 138-140)**

462: Doesn’t GOF need validation?

**As stated above, we include a validation of the goodness-of-fit values in the Supporting Information now.**
470: The main verb is missing. **The ‘that’ was wrongly added and made the sentence unclear, thank you. (line 507-508).**
503: “probability…increased” has a strong, quantitative connotation, which is at present not established in the manuscript. **We hope you also find this to be better established with the included examples on artificial data now.** **(S1 Supporting Information, Table 2)**
552-554: I believe mentioning Julia Hjulia yland Bruno’s work here would be quite relevant.

**That is true, thank you for making us aware of this, we had already read the work and for reasons unknown not yet included it (lines 593-595).**
Figure 1: I wonder whether the authors should reference here the Frontiers paper by Ravagnani Bowling and Fitch containing a similar schematic. **Thank you, we now state that the schematic was adjusted from the mentioned paper. (line 170)**
Figure 7: Why is the cutoff at exactly 1 sec?

**The cut off does not need to be at exactly 1 sec. 1 sec was supposed to be an example of short sequences, as this has an important impact on the frequency resolution of the FFT. But since it is not a clear cut off, we changed the workflow to ‘short sequences (i.e. < 1 sec)’ and ‘long sequences’.**
References: There are a few issues/typos, please check them. For instance, ref #14: Celma & Toro were authors not editors. Also, a paper describing the work in that abstract recently appeared in J of Comparative Psychology. **The references were carefully checked again. We hope all issues and typos were cleared. Thank you.**

Reviewer #2: Review of manuscript PCOMPBIOL-D-19-02059, ‘Method comparison for rhythm analysis of complex animal vocalizations’.

This manuscript focuses on an important subject in computational biology, and the authors are experienced in the topic. However, I find that the presentation is a bit too focused on the several selected species, and this concentration will prevent the paper from attaining its stated goal : showing biologists – who may not have considerable experience with acoustic signals and rhythm – how they might best conduct their analyses.

**Thank you for your valuable comments on our manuscript. In contrast to your view, the aim of this paper was the focus on actual biological data, as a paper on artificial data already exists (Ravignani and Norton, 2017). Nevertheless, we are happy to now include some artificial data, to better illustrate key innovations of the manuscript at hand (lines 270 – 277 and Supporting Information). Also we included a glossary to prevent confusion of terminology.**
Main points
1 It would be more helpful to begin with an overview of rhythm in acoustic signals and introduce the key features that can be identified among many animal species. Some attention should be paid to ‘nested’ parameters ; e.g. calls that are comprised of syllables, which in turn are comprised of pulses, each one of these units having a rhythm, unit duration, and inter-unit gap (or onset-to-onset interval). Ideally, this framework would be established without reference to the specific terms (e.g. syllables, pulses), as different authors tend to use their own definitions.

**Thank you for this thought. We appreciate the reference to nested parameters, but as you state, certain calls are comprised of syllables, that have their own rhythm. We only provide the framework to analyse this, for what units and subunits of data this could be done for is not the focus of this work. Nevertheless, we now mention nested parameters and the possibility to use the analysis on various levels in the manuscript (lines: 158-161). We already tried to refrain from using specific terms such as syllables and pulses by using the term ‘element’. This is explained in the text (line 126) and in the newly established glossary (line 105). We carefully re-read the manuscript to be sure to be consistent in the use of that term.**
2 The authors note the potential importance of acoustic rhythm in biology, but it would be helpful to specify the different fields of biology where measurement of rhythm could be valuable and what aspects might be most important to measure. For example, rhythm could be critical for assessing species identity, gender or developmental stage, an individual’s social status, and individual identity. These several factors might demand different types of analysis in order to extract the most relevant information.

**As we try to illustrate with different examples, this is also one of our main goals, to note the potential in these methods (lines 13-15, 62-77 ). We agree that the information on temporal structure might be informative for questions on species identification (example line 73-75), or individual identity (lines 108-112). You are right that it might also be informative for developmental stage or social status. So far, there is no evidence of that; in fact, in a previous study we found that in the analysed bat *Saccopteryx bilineata* the rhythm does not encode developmental stage (Burchardt et al, 2019). Nevertheless, this is a valuable aspect of the analysis. More work is needed on what questions to analyse, and what paths to go with the methodology we now established as a tool. We therefore hope that researchers can be guided reasonably by our workflow. A future work of us will be a paper on the outlook of these methods and what questions to answer with exactly what kind of dataset as well as the implications for qualitative versus quantitative analysis in the framework of rhythm analysis in animal communication.**

3 More attention should be paid to using terms in a more rigorous way and to being consistent with their use in other fields of science. For example, ‘beat’ has a very specific meaning in physics (beat frequency = difference between two different frequencies), whereas the authors are using the term in a loose manner to refer to animal calls. While ‘beat’ may have been used in this loose fashion by workers studying a particular group of animals, it is not at all general and will cause confusion. Many other examples exist in the manuscript. Another issue is the relationship between rhythm and periodicity, wherein the authors assume that rhythms always entail a certain period of repetition of the relevant units. However, workers studying human language often recognize a ‘language rhythm’ that does not depend on any particular period. This issue needs careful consideration, particularly when dealing with mammalian vocalizations.

**This is a very important point and we highly appreciate the comment! We were not aware that even ‘beat’ has such a specific meaning in another context. To clarify any concrete connotation, we added a glossary including some of the key terms used in the paper (line 105). In that we also explain our use of “rhythm” and “periodicity”. As you state, the use of these needed careful consideration.** **We include statements on this at various points in the text now (lines 56-61,82-94,138-140)**. **The use of the term ‘rhythm’ is highly controversial in this context, and we are aware of that. Which is why we totally agree it is necessary to specifically define our use. We hope we do that better now.**

4 The manuscript is fraught with an excessive amount of jargon, a feature that is likely to deter the generalist reader interested in applying rhythm analysis for the first time. Some jargon is, of course, necessary, but these specialized terms begin very early in the manuscript and dominate it.

**To enable a broader audience to understand the manuscript, we included a glossary – as stated above (line 105). It includes terms such as ‘vocalization’, ‘periodicity’ and others. Also we carefully re-read the manuscript trying to avoid jargon and technical terms where ever possible and explaining them in case they can not be avoided. (i.e. lines 73-74). Furthermore we made sure to use the explained terms in a consistent manner.**

5 Using examples of actual vocalizations is helpful, but they should be presented only to show what sorts of analyses are applicable in particular cases. However, the authors have built their manuscript around several vocalization examples, and the reader is not left with a general idea of where to use method a, method b, etc. And, a given method may reveal certain critical features in vocalization a while another method reveals other features in the same vocalization ; i.e. the application of several methods may be best. More importantly, the several vocalization examples presented by the authors represent a very small portion of the range of vocalization rhythms that biologists can encounter.

**We thank the reviewer for this assessment. We agree that the application of several methods can reveal different aspects of a temporal structure and is therefore helpful to get a full picture. Nevertheless, by establishing the workflow, we tried to state which methods might not be applicable at all for certain datasets and which methods to start with. We are aware that the used datasets, even though already quite variable, only make up the smallest part of possible datasets. Therefore we had already included some more examples and which ‘route’ to use for that specific example in the workflow (lines 548-556 ). To stress that, we added the examples to Figure 7. Furthermore, we now include artificial data that we analysed the same way as our original datasets, to give a more detailed picture of what results might look like. We also want to stress again, that an elaborate paper on several of the used methods and their results in artificial data already exists (Ravignani & Norton, 2017), which is why we did not focus on that here.**

6 Studying acoustic rhythm is a fundamental aspect of animal behavior and physiology, but animals often exhibit their rhythms in the company of conspecifics and thereby generate rhythm interactions. Some mention / treatment of measuring rhythm interaction would be helpful.

**This is a first step in establishing a framework for the analysis of rhythm in animal acoustic signals. As much as we would like to be able to analyse rhythm in interactions in a meaningful and comparative way, this is not where we are with the methods yet. Some good models exist, where the interactions can be studied, and we are looking forward to examples on that. This study focuses on non-interactive communication, which is why we cannot include this here.**

Minor point
7 A simple method that often works to identify rhythm periodicity is harmonic analysis : An individual may call with a given free-running (endogenous) rhythm period, but owing to various internal and external stimuli he/she can miss one or several calls at times. By arranging the individual’s inter-call intervals in time bins, periodicity is revealed by the presence of most inter-call intervals in bin X, a smaller number of intervals in bin 2X, a yet smaller number in bin 3X, etc.

**This sounds much like the visual assessment of IOI histograms, were you can also see the presence of most IOIs in bin X, it might be a complementary analysis, thank you for pointing us to this similarity.**
